# Sentinel Lymph‐Node Biopsy Guided Neck Dissection Versus Elective Neck Dissection in the Management of Early‐Stage Oral Cancer—A Cost‐Utility Analysis

**DOI:** 10.1002/cam4.71571

**Published:** 2026-02-05

**Authors:** Shivakumar Thiagarajan, Shweta Sharda, Yashika Chugh, Nidhi Gupta, C. S. Pramesh, Shankar Prinja

**Affiliations:** ^1^ Department of Surgical Oncology Tata Memorial Hospital, Parel, Homi Bhabha National Institute Mumbai India; ^2^ Department of Community Medicine & School of Public Health Post Graduate Institute of Medical Education & Research Chandigarh India; ^3^ Department of Radiation Oncology Government Medical College and Hospital Chandigarh India

**Keywords:** cost‐effectiveness analysis, elective neck dissection, occult metastasis, oral squamous cell carcinoma, sentinel lymph node biopsy

## Abstract

**Objectives:**

This study aims to assess the incremental cost per quality‐adjusted‐life‐year (QALY) gained in treating patients with early oral squamous cell carcinoma (OSCC) using sentinel lymph node biopsy (SLNB) guided neck dissection.

**Methods:**

A Markov model was created to simulate disease‐free survival, recurrence, and overall survival in a hypothetical cohort of patients with early OSCC in India. Three groups were assessed: Group I—SLNB‐guided neck dissection, Group II—elective neck dissection (END) alone, and Group III—END with frozen section (FS). Costs and QALY were assessed using a payer's perspective, lifetime horizon, and 3% discount, and incremental cost utility ratios (ICUR) were computed. Interventions with ICUR less than one‐time gross domestic product (GDP) per capita were considered cost‐effective. Both one‐way and probabilistic‐sensitivity analyses were conducted to examine model uncertainty.

**Results:**

In comparison to Group II and Group III, Group I incurs additional costs of INR 5564 (US$ 67) and INR 2507 (US$ 30) per patient, respectively, and results in an incremental gain of 0.31 and 0.33 additional QALYs, respectively, over a lifetime horizon. The ICURs for Group I versus Group II and Group III are INR 8088 (US$ 97) and INR 16,709 (US$ 200), respectively. At a threshold of one‐time per‐capita GDP, SLNB demonstrates a 94% probability of being cost‐effective.

**Conclusion:**

SLNB‐guided neck dissection is a cost‐effective strategy for management of early OSCC in India. Our findings support inclusion of SLNB‐guided neck dissection in the Indian Government's insurance program.

## 1 Introduction

Oral squamous cell carcinoma (OSCC) is among the most common cancers globally, with a reported incidence exceeding 350,000 and approximately 177,000 deaths annually in the year 2018 [1]. With an age‐standardized rate (ASR) of 4.1 per 100,000 population, OSCC ranks as the seventeenth most common cancer globally. In India, it is the second most common cancer overall, and is the most prevalent cancer among males [2].

The management of OSCC is primarily surgical, the type and extent of which is decided by the stage of the disease. Nodal metastasis is an important prognostic factor in patients with OSCC. Presence of nodal metastasis decreases survival by 50% [3]. In the presence of clinically and radiologically metastatic neck nodes, a neck dissection becomes an integral part of the surgical treatment plan [4]. In patients with clinically and radiologically nonmetastatic nodes (cT_1_T_2_N_0_), the rate of occult nodal metastasis is up to 30% [5]. In view of this, elective neck dissection (END) is advocated for all patients with cT_1_T_2_N_0_ OSCC [4]. However, it is important to take into account that occult metastasis is observed in only approximately one‐third of these patients. END done for early OSCC entails removal of lymph nodes of level I–III which may be associated with morbidity, in particular shoulder morbidity (9%–25%) [6]. These factors can have a detrimental impact on the overall health‐related quality of life (HRQoL) of these patients, further emphasizing the need to carefully consider the benefits and potential drawbacks of END in _c_T_1_T_2_N_0_ OSCC. In view of these considerations, alternative treatment strategies for the neck have been tried that can accurately assess occult metastasis and guide the choice of surgical intervention needed. Sentinel lymph node biopsy (SLNB) is one such recent advancement.

The concept of sentinel lymph nodes (SLN) is based on the assumption that cancer cells will first metastasize from the primary site to a single node or a cluster of nodes, known as the sentinel nodes, before spreading to the remaining lymph nodes in the region. A recently published meta‐analysis of 12 studies involving 10,583 patients concluded that there is no statistically significant difference in the overall survival (OS) between SLNB guided neck dissection and END, and that both strategies have similar prognostic value among patients with early stage clinically node‐negative OSCC [7].

The management approach in patients with cT_1_T_2_N_0_ OSCC depends not only on the clinical outcomes, but also on the associated costs of treatment. The study by Govers et al. [8] used a Markov decision model to assess and compare the cost‐effectiveness of five different procedures including SLNB‐guided neck dissection and END from a health‐system perspective in the Netherlands. The study concluded that over a 5‐year time horizon, SLNB with an incremental cost of €3356 per quality adjusted life year (QALY) gained and willingness to pay (WTP) threshold of €80,000 is a cost‐effective strategy as compared to END. Another decision model assessed the cost‐utility of four strategies (ultrasound‐guided fine needle aspiration cytology [USG FNAC]; SLNB; USG FNAC and, if negative, SLNB; END) from a hospital perspective for the detection of occult lymph node metastases in cT1–T2N0 oral cancer in the Netherlands [9]. For a 5 years and 10 years time horizon, the study findings favored SLNB as it yielded the highest QALYs while incurring limited incremental costs.

Given the evidence, it is pivotal to note that the existing literature on SLNB primarily includes studies conducted in developed countries [8, 9] where healthcare standards and resources differ substantially from those in low‐and‐middle‐income countries (LMICs) [10]. These studies might not directly mirror the actual situations and difficulties faced by LMICs, such as India. In a survey conducted among surgeons treating OSCC in India, only 15.1% of the respondents used SLNB for early OSCC. Additional cost with the use of SLNB was considered one of the major deterrents by 71.9% of the respondents [11]. Moreover, the values assigned to health conditions are different, and the WTP thresholds in affluent nations tend to be higher than those observed in LMICs [12, 13, 14]. These factors can influence the generalizability of the conclusions from existing economic evaluations within LMIC environments. Given these limitations, there is a need for context‐specific evidence that can provide insights into the incremental costs per QALY gained for managing patients with _c_T_1_T_2_N_0_ disease with SLNB‐guided neck dissection in the Indian scenario. Our study aims to address this gap by evaluating the cost‐effectiveness of SLNB‐guided neck dissection versus END in the Indian context to guide decision‐making and help optimize the management of patients with _c_T_1_T_2_N_0_ OSCC.

## 2 Methods

### 2.1 Overview of Analysis

The Indian clinical practice consensus guidelines for the management of localized oral cancer also suggest that primary tumor resection should be followed either by END in all patients or SLNB‐guided ND in those patients who test positive for nodal metastasis in the SLN [4]. While the END approach involves surgical excision of Level I‐III lymph nodes in all the patients, the SLN approach involves a series of steps to be performed during the primary surgery. Firstly, the surgeon locates the SLNs using perioperative peritumoral injections of a radiotracer, followed by lymphoscintigraphy using single photon emission computed tomography/CT (SPECT‐CT) imaging. Next, a handheld gamma probe is used intraoperatively to detect the SLNs which are then surgically removed. This SLN is sent for frozen section (FS) analysis for the presence of metastasis, and if found positive, a modified neck dissection (MND) is performed. If the SLN is negative for metastasis, no further surgery is needed. These nodes are further subjected to a detailed analysis and reported in the final histopathological report. If metastasis is observed in the final histopathological analysis, the patient is re‐admitted within one month to receive MND [15, 16].

In the present study, the health outcomes were evaluated in terms of life‐years (LY) and QALYs, which account for the impact of both quantity and quality of life. To assess the change in costs and outcomes as a result of alternative treatment strategies and identify the most cost‐effective option, the incremental cost‐utility ratio (ICUR) was calculated [17, 18]. As this study relied on publicly accessible secondary data, ethical approval was not required. We used a one‐time per‐capita Gross Domestic Product (GDP) value specific to India for the year 2023–24 as the threshold for determining cost‐effectiveness, that is, INR 2,11,725 (US$ 2530) [19, 20, 21]. Finally, the incremental net monetary benefit (INMB) was calculated as the product of incremental effects (e.g., QALYs gained) and willingness‐to‐pay, minus incremental costs. A positive INMB indicates that the strategy is cost‐effective at the specified WTP threshold, making it a worthwhile investment compared to the alternative strategy.

### 2.2 Model Overview

#### 2.2.1 Treatment Groups

*Group I*: SLNB‐guided neck dissection using intraoperative FS analysis comprised the intervention group. The most used agent for SLNB in India is methylene blue followed by Technetium 99 (99Tc) sulfur colloid [11]. The sensitivity and negative predictive value (NPV) of SLNB with FS analysis are assumed to be 81% and 93%, respectively [22]. Based on the diagnostic accuracy of SLNB with FS analysis compared to histopathological examination, patients were further categorized as true positives (TP), false positives (FP), true negatives (TN), and false negatives (FN). TP patients are those who truly have positive nodes as per both the perioperative SLNB and postoperative histopathological assessment. FP patients are those who do not have positive nodes on histopathological examination but whose SLNB test result is positive. Patients are categorized as TNs if both the histopathological examination and SLNB result is negative. Those patients who tested negative on SLNB but turned out to be positive on subsequent postoperative histopathological assessment were labeled as FN. However, those patients who tested negative on both perioperative SLNB and subsequent postoperative histopathological examination but subsequently report a positive histopathological report on follow‐up visits are considered as “recurrence.”

*Group II*: The comparator group included patients undergoing selective neck dissection (SND) procedure that involved removal of lymph nodes at level I–III (END alone).

*Group III*: As the ideal treatment of a node positive neck is MND (removal of Level I–V nodes), it is believed that intraoperative FS analysis can be used to determine the neck metastasis and change the surgical strategy to a MND if needed. To account for these variations that can be considered in the END group, we assumed END with intraoperative FS analysis (END with FS) as another comparator group (denoted as *Group III*). In patients belonging to *Group III*, the patients that test positive to the intraoperative FS analysis are further subjected to MND, and the rest of the patients undergo SND alone. The sensitivity and NPV of END with FS analysis were assumed to be 84% and 93% respectively [22].

Patients in the three groups who have confirmed metastatic nodes on postoperative histopathological examination are further subjected to adjuvant therapy, which included radiotherapy (RT) either with or without chemotherapy (CT), based on standard treatment guidelines. Rest of the patients are followed up routinely as per guidelines [4, 23].

Our model calculated lifetime costs and health benefits for each of the three treatment groups. We then ranked the treatment groups in ascending order of their effectiveness, and each group was compared with the precedent to draw conclusions regarding cost‐effectiveness [24].

### 2.3 Model Structure

**FIGURE 1:**
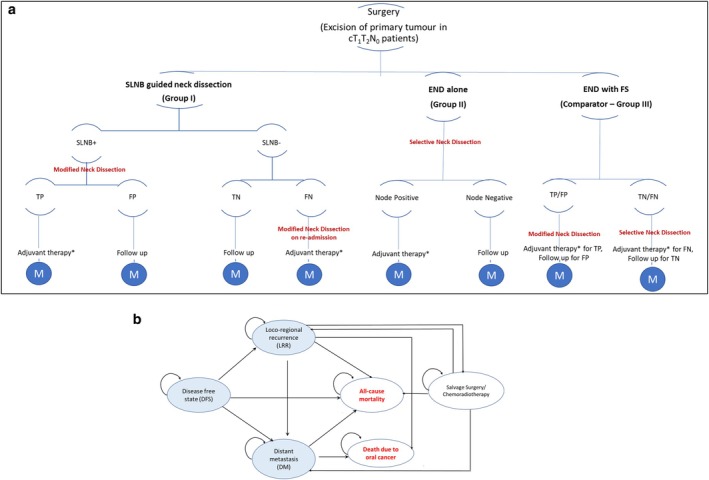
(a) Decision model showing various treatment modalities for management of early‐staged OSCC patients. (b) Markov‐model showing transition among different health states. −, negative; +, positive; END, elective neck dissection; FN, false negative; FP, false positive; FS, frozen section; M, Markov model; SLNB, sentinel lymph node biopsy; TN, true negative; TP, true positive. *Adjuvant therapy comprises of radiotherapy with or without chemotherapy as per standard treatment guidelines.

Considering the median age of presentation of OSCC to be 52 years, we assumed a hypothetical cohort of 50‐year‐old Indian patients clinically and radiologically diagnosed with T_1_T_2_N_0_ OSCC [25]. A decision tree (Figure 1a) integrated with a Markov model (Figure 1b) was created in Excel Windows 2010 (Microsoft, Redmond, WA, USA) to evaluate the cost‐effectiveness of the three treatment strategies for the management of OSCC [26]. The decision tree begins with the early staged OSCC patients who have been all subjected to the excision of the primary tumor. Next, the decision is to follow one of the three treatment procedures: SLNB (Group I), END alone (Group II), or END with FS (Group III). Once the respective surgical procedure is completed in the three groups, the patient enters the Markov model and is followed up for a lifetime horizon.

Based on the natural history of oral cancer progression as stated in the study by D'Cruz et al. [27] we modeled six health states including disease‐free survival (DFS), loco‐regional recurrence (LRR), distant metastasis (DM), and those that undergo salvage surgery after LRR. Death due to oral cancer and all‐cause mortality were the terminal states in the model. After the primary treatment involving surgical excision of the tumor with or without neck dissection as indicated, all patients achieved DFS. The cycle length was assumed to be one year. After each cycle, the patient could either remain in the same health state or move to a different health state. The probability of progression to LRR or DM from the DFS depends on the initial nodal status and was derived from a retrospective study that utilized the data of 595 OSCC patients to develop a DM prediction model [28] The rate of disease progression from LRR to DM was assumed to be similar in both node‐positive and node‐negative patients [28]. Further, the recurrence rate in salvaged patients was derived from a retrospective study conducted on 377 patients who had recurrent squamous cell carcinoma of the head and neck [29]. The probability of death due to oral cancer from the LRR state was derived from a previous study that has reported an oral cancer‐specific mortality rate of 85% in 2 years [30]. The probability of survival after development of distant metastasis was considered to be 7.5 months based on the results reported by a recent study among head and neck cancer patients with distant metastasis [31]. The risk of all‐cause mortality from any unrelated illness was obtained from the Indian sample registration survey (SRS) life tables [32].

A payer's perspective was employed to comprehensively assess the economic impact of the intervention within the financial context of the payer. The payer in our context is assumed to be the national insurance program in India—*Ayushman Bharat Pradhan Mantri‐Jan Arogya Yojana (PM‐JAY)*. Indirect costs resulting from productivity losses were not taken into account in the analysis. This follows from the perspective of the economic evaluation of healthcare programs and is also in agreement with the guidelines endorsed by the Indian HTA agency, which recommends exclusion of indirect costs in the analysis [18, 20, 33]. To ensure consistency with Indian HTA reference case, both future costs and consequences were discounted at a rate of 3% [20, 33]. The model employed an annual Markov cycle, and the outcomes were reported for a lifetime horizon. Half‐cycle correction was applied [34].

### 2.4 Clinical Parameters and Utility Values

The diagnostic accuracy of SLN biopsies was obtained from the pooled analysis of sensitivity and specificity estimates of seven studies on 457 patients of head and neck cancer [35]. A recent meta‐analysis of five randomized controlled trials and 34 retrospective studies on patients with cN_0_T_1_T_2_ OSCC who underwent END reported the pooled rate of occult metastasis weighted by study size to be 23% [36]. The diagnostic accuracy estimates of Group I as well as Group III were used to derive the proportion of TP, FN, FP, and TN in the two groups respectively [22].

To estimate the utility values for each Markov health state, we used the data collected as part of a multi‐centric cross‐sectional Indian study aimed at developing a National Cancer Database of Costs and Quality of Life (CaDCQoL), in which primary data was collected from patients with cancer at seven leading cancer centres from six states across the country [37, 38]. The CaDCQoL includes data on HRQoL using the EQ‐5D‐5L instrument for 687 patients with oral cancer [38]. Of these patients, 87 were identified as being in the distant metastasis health state (stage IV‐C), and they reported a utility score of 0.505. For other health states (DFS after primary tumor resection, DFS after neck dissection, LRR, and salvage), the utility values were derived in two steps. Firstly, utility scores from published literature were used to compute a gradient for each of these health states (DFS after primary tumor resection, DFS after neck dissection, LRR, and salvage) relative to the DM state [30]. Next, this gradient was applied to the utility estimates of DM as estimated from CaDCQoL to obtain the utility values for all other health states for the Indian population.

For the TN patients who undergo SLNB alone, without any neck dissection, the utility value computed for the DFS state after primary tumor resection was applied. Next, to account for the impact of the extent of neck dissection on the HRQoL, we applied separate estimates of utility for MND and SND to the respective neck‐dissection patients [30, 39]. The health state utility values for SND and MND were applied for a period of five years, after which the health state utility of the DFS state was assumed to resolve back to that of neck dissection alone, irrespective of the type of neck dissection performed [40].

### 2.5 Cost Parameters

For the DFS state in Group I patients, the SLNB procedure related costs were considered. Additionally, for the SLNB TP, FP, and FN patients in Group I, the costs related to the MND procedure were also considered. For the patients in Group II and Group III, costs related to the SND and MND procedures were considered, as applicable. For all the histopathologically node positive patients, cost for adjuvant RT with or without CT was also considered apart from the surgical costs, as per standard treatment guidelines. For the treatment costs in the LRR state, it was assumed that patients would be either subjected to RT with/without CT or undergo salvage surgery, and the costs were considered accordingly. For patients who develop DM, palliative care treatment costs were considered as per guidelines [4]. Apart from these surgical, medical, and radiation treatment cost‐packages, the cost of routine investigations including complete blood cell count, liver and kidney function test, serum electrolytes, and radiology were assumed to be conducted at the follow‐up visits as indicated. We did not include the cost of resection of the primary tumor as all patients in our model were subjected to primary surgery, and thus would not lead to any difference in costs incurred in comparative scenarios.

We used the Government funded national health insurance scheme—*PM‐JAY's* provider payment rates, as reported in its recent health benefit package (HBP) 2022, for the surgical, medical and radiation costs. These package rates are inclusive of all pre‐ and post‐hospitalization costs including consultation, diagnostic tests, medicines, and follow‐up investigations up to 15 days after discharge from the hospital for the same surgery. Indian cities are classified into three tiers based on population size, infrastructure development, economic growth, and overall quality of life. Accordingly, the *PM‐JAY* offers tier‐based differential package rates for various secondary care and tertiary care procedures [41]. In our model, the base case treatment costs for the surgical, medical and radiation oncology packages including neck dissection (modified/selective), SLNB, CT and RT were computed as the weighted average of package rates from the Tier‐1, 2 and 3 cities. This weighting was based on the number of claims settled for the oncology HBPs across the top 10 hospitals stratified according to their city type [42]. Notably, these hospitals serve more than 60% of cancer patients in India [42]. For the post‐discharge routine follow‐up investigations which are not a part of the *PM‐JAY* health benefit packages, we used the Central Government Health Scheme (CGHS) cost estimates [43]. All costs were expressed in Indian Rupee (INR) and converted to US Dollars (US$) using the average exchange rate of 1 US$ = INR 83.68 for the year 2024 [44]. All the input parameters have been detailed in Table 1.

**TABLE 1:** The estimates and data sources of various input parameters used in the model.

Input parameter	Base estimate	Lower limit	Upper limit	Distribution	Source
Median age of patients	50	—	—	Fixed	[25]
Rate of occult metastasis	0.23	0.2	0.3	Beta	[36]
Sensitivity (SLNB)	0.81	0.77	0.85	Beta	[22]
Negative predictive value (SLNB)	0.93	0.88	0.98	Beta	[22]
Sensitivity (END + FS)	0.84	0.80	0.88	Beta	[22]
Negative predictive value (END + FS)	0.93	0.88	1.00	Beta	[22]
Utility values					
Utility SND without morbidity	0.862	0.82	0.91	Beta	[30, 37]
Utility SND with morbidity	0.747	0.71	0.78	Beta	[30, 37]
Utility MND without morbidity	0.821	0.78	0.86	Beta	[30, 37]
Utility MND with morbidity	0.711	0.68	0.75	Beta	[30, 37]
DFS utility after primary tumor resection	0.891	0.85	0.94	Beta	[30, 37]
DFS utility after neck dissection	0.819	0.78	0.86	Beta	[30, 37]
Utility LRR	0.659	0.63	0.69	Beta	[30, 37]
Utility DM	0.508	0.48	0.53	Beta	[37]
Utility salvaged	0.264	0.25	0.28	Beta	[30, 37]
Transition probabilities (annual)					
DFS TO LRR (node positive)	0.065	0.062	0.069	Dirichlet	[28]
DFS TO LRR (node negative)	0.043	0.041	0.045	Dirichlet	[28]
DFS TO DM (node positive)	0.0136	0.013	0.014	Dirichlet	[28]
DFS TO DM (node negative)	0.004	0.004	0.005	Dirichlet	[28]
LRR to DM	0.076	0.072	0.08	Dirichlet	[28]
LRR to salvage (dissected neck)	0.083	0.079	0.087	Dirichlet	[29]
LRR to salvage (no neck dissection)	0.146	0.139	0.154	Dirichlet	[29]
LRR to death due to oral cancer	0.613	0.582	0.643	Dirichlet	[30]
Salvage to LRR (dissected neck)	0.136	0.129	0.143	Dirichlet	[29]
Salvage to LRR (no neck dissection)	0.115	0.109	0.121	Dirichlet	[29]
Salvage to DM (dissected neck)	0.032	0.030	0.033	Dirichlet	[29]
Salvage to DM (no neck dissection)	0.030	0.029	0.032	Dirichlet	[29]
51–55 years all cause mortality	0.008	—	—	Fixed	[32]
56–60 year all cause mortality	0.013	—	—	Fixed	[32]
61–65 years all cause mortality	0.019	—	—	Fixed	[32]
66–70 years all cause mortality	0.029	—	—	Fixed	[32]
71–75 years all cause mortality	0.151	—	—	Fixed	[32]
76–80 years all cause mortality	0.066	—	—	Fixed	[32]
80+ years all cause mortality	0.103	—	—	Fixed	[32]
Cost parameters INR (US $)					
Complete blood cell count	INR 155 (US$ 1.97)	INR 124 (US$ 1.58)	INR 186 (US$ 2.37)	Normal	[43]
Liver function test	INR 259 (US$ 3.29)	INR 207 (US$ 2.64)	INR 311 (US$ 3.95)	Normal	[43]
Renal function test	INR 259 (US$ 3.29)	INR 207 (US$ 2.64)	INR 311 (US$ 3.95)	Normal	[43]
Serum electrolytes	INR 460 (US$ 5.85)	INR 368 (US$ 4.68)	INR 552 (US$ 7.02)	Normal	[43]
Serum thyroid stimulating hormone	INR 104 (US$ 1.32)	INR 83 (US$ 1.06)	INR 125 (US$ 1.59)	Normal	[43]
Cisplatin 40 mg/m^2^	INR 2900 (US$ 37)	INR 2600 (US$ 33)	INR 3100 (US$ 39)	Gamma	[41]
Cisplatin 100 mg/m^2^	INR 11,950 (US$ 152)	INR 10,800 (US$ 137)	INR 12,800 (US$ 163)	Gamma	[41]
2D external beam radiotherapy	INR 13,390 (US$ 170)	INR 12,100 (US$ 154)	INR 14,300 (US$ 182)	Gamma	[41]
3D‐CRT	INR 25,540 (US$ 325)	INR 23,100 (US$ 294)	INR 27,300 (US$ 347)	Gamma	[41]
IMRT	INR 85,050 (US$ 1082)	INR 77,000 (US$ 979)	INR 91,000 (US$ 1158)	Gamma	[41]
High end radiological diagnostic	INR 5560 (US$ 71)	INR 5000 (US$ 64)	INR 5800 (US$ 74)	Gamma	[41]
Paclitaxel 175 mg/m^2^	INR 14,380 (US$ 183)	INR 13,000 (US$ 165)	INR 15,400 (US$ 196)	Gamma	[41]
Paclitaxel + Carboplatin 175 mg/m^2^	INR 17,540 (US$ 223)	INR 16,000 (US$ 204)	INR 18,200 (US$ 232)	Gamma	[41]
Basic supportive care	INR 10,215 (US$ 130)	INR 9900 (US$ 126)	INR 10,350 (US$ 132)	Gamma	[41]
Sentinel lymph node biopsy	INR 28,670 (US$ 365)	INR 25,900 (US$ 330)	INR 25,900 (US$ 330)	Gamma	[41]
Modified neck dissection	INR 36,870 (US$ 469)	INR 33,300 (US$ 424)	INR 39,900 (US$ 508)	Gamma	[41]
Selective neck dissection	INR 26,040 (US$ 331)	INR 23,500 (US$ 299)	INR 28,200 (US$ 359)	Gamma	[41]
Salvage surgery	INR 60,550 (US$ 770)	INR 54,600 (US$ 695)	INR 65,600 (US$ 835)	Gamma	[41]
Frozen section	INR 8500 (US$ 108)	INR 5000 (US$ 64)	INR 12,000 (US$ 153)	Gamma	Market survey

Abbreviations: 2D, 2 dimensional; 3D‐CRT, 3‐dimensional conformal radiotherapy; DFS, disease‐free‐survival; DM, distant metastasis; END, elective neck dissection; FS, frozen section; IMRT, intensity modulated radiotherapy; LRR, loco‐regional recurrence; MND, modified neck dissection; SLNB, sentinel lymph node biopsy; SND, selective neck dissection.

### 2.6 Sensitivity Analysis

One‐way sensitivity analyses were performed, where individual parameters were varied one at a time, keeping the other parameters constant at their baseline values [45]. The minimum and maximum value to which the parameter's baseline value was varied to was determined from the lower and upper limits of the 95% confidence interval (95% CI) of the particular parameter. Alternatively, if these bounds were unavailable, we utilized a ±20% margin, or resorted to the range between the minimum and maximum values documented in the literature [46]. A tornado diagram was constructed to visually represent the impact of parameter variations on the baseline ICUR.

Secondly, a probabilistic sensitivity analysis (PSA) was also conducted to account for the impact of joint uncertainty of parameters which were given a theoretical probability distribution [47]. Monte Carlo simulations were employed to run 1000 model iterations. The PSA outcomes were presented using cost‐effectiveness (CE) planes and cost‐effectiveness acceptability curves (CEAC) [48, 49].

The study findings are reported as per the Consolidated Health Economic Evaluation Reporting Standards (CHEERS) guidelines [50]. Additionally, we also present our findings in accordance with the author reporting guidelines outlined in the Indian Health Technology Assessment Quality Appraisal Checklist (HTA‐QAC), which is employed for the evaluation of studies conducted by the HTA body in India (HTAIn) [51].

## 3 Results

### 3.1 Valuation of Costs

The lifetime cost per person in Group I is estimated at INR 181,520 (95% CI: INR 171,081 to INR 192,897) [US$ 2169 (95% CI: US$ 2045 to US$ 2305)]. The patients in Group II incurred an average lifetime cost of INR 179,012 (95% CI: INR 168,391 to INR 190,580) [US$ 2139 (95% CI: US$ 2012 to US$ 2278)] per person, while that in Group III incurred INR 175,956 (95% CI: INR 165,489 to INR 187,273) [US$ 2103 (95% CI: US$ 1978 to US$ 2238)].

When compared to Group II, Group I incurs additional lifetime costs of INR 2735 per person (95% CI: ‐ INR 3774 to INR 8837); [US$ 33 (95% CI: ‐ US$ 45 to US$ 106)]. The incremental cost of Group I versus Group III was INR 5757 per person (95% CI: ‐ INR 349 to INR 11,414); [US$ 69 (95% CI: ‐ US$ 4 to US$ 136)].

### 3.2 Valuation of Outcomes

Our findings show Group I to be the most effective treatment modality followed by Group II and III. Over a lifetime horizon, both Group II and III yielded similar LYs, that is, 10.17 (95% CI: 10.01–10.36) which were lower than that observed in Group I, that is, 10.25 (95% CI: 10.12–10.39). Similarly, the QALYs per person were also noted to be highest for Group I [8.43 (95% CI: 8.41–8.72) QALYs], compared to 8.12 (95% CI: 7.81–8.43) QALYs for Group II and 8.097 (95% CI: 7.79–8.41) QALYs for Group III. Group I yielded an additional 0.32 QALYs (95% CI: −0.05 to 0.68) and 0.34 QALYs (95% CI: −0.02 to 0.69) in comparison to Group II and III, respectively.

### 3.3 Cost‐Effectiveness

To evaluate cost‐effectiveness among the three interventions, the treatment Groups I, II, and III were ranked based on the ascending order of their Quality‐Adjusted Life Years (QALYs) (Table 2). ICURs were then calculated for each strategy compared to the previous alternative. When comparing Group II with Group III, the incremental cost per QALY gained is INR 118,473 (955 CI: INR 62,773 to INR 451,397) [US $ 1416 (95% CI: ‐US$ 750 to US $5394)]. The ICUR for Group I versus Group II and III is INR 7672 (95% CI: ‐INR 69,909 to INR 108,134) [US $ 92 (95% CI: ‐US$ 835 to US $1292)] and INR 15,743 (95% CI: ‐INR 53,261 to INR 145,014) [US $ 188 (95% CI: ‐US$ 637 to US $1733)], respectively.

**TABLE 2:** Costs and health outcomes for the three treatment groups at a lifetime‐horizon.

Treatment groups	Cost per person	Incremental cost^a^	QALYs per person	Incremental QALYs^a^	Incremental cost per QALY gained^a^	Incremental net monetary benefit^a^
Group III	INR 175,956 (US $2103)	—	8.097	—	—	—
Group II	INR 179,012 (US $2139)	INR 3056 (US $37)	8.12	0.023	INR 132,888 (US $1588)	INR 351 (US $4)
Group I	INR 181,520 (US $2169)	INR 2507 (US $30)	8.43	0.31	INR 8088 (US $97)	INR 64,430 (US $770)

*Note:* This table represents the results of dominance analysis which is based on the deterministic results; the treatment groups are ordered in terms of increasing effects (QALYs). Group I: Sentinel Lymph Node Biopsy guided Neck Dissection; Group II: Elective neck Dissection Alone; Group III: Elective Neck Dissection with Frozen Section Analysis.

Abbreviations: END, elective neck dissection; QALYs, quality‐adjusted‐life‐years; SLNB, sentinel lymph node biopsy.

^a^ Compared to the preceding treatment group.

Considering the relatively short‐term impact of the surgical modality on patients' HRQoL, we conducted a scenario analysis to present the model's outcomes at both 5‐ and 10‐year time horizons. Detailed findings from this analysis can be found in the Supporting Information.

### 3.4 Sensitivity Analysis

Deterministic sensitivity analysis demonstrated that the ICUR values were most sensitive to variations in the DFS utility after primary tumor resection, rate of occult metastasis, and the Negative Predictive Value of SLNB. The tornado diagram showing the results of the deterministic sensitivity analysis at a lifetime scenario is presented in Figure S1. Additionally, we also performed a Threshold analysis to assess the breakpoint rate of occult metastasis until which SLNB guided neck dissection strategy remains cost‐effective. It was observed that up to 60% of occult metastasis rate, SLNB can be considered a cost‐effective strategy at the current one‐time GDP per capita threshold in India (Supporting Information).

**FIGURE 2:**
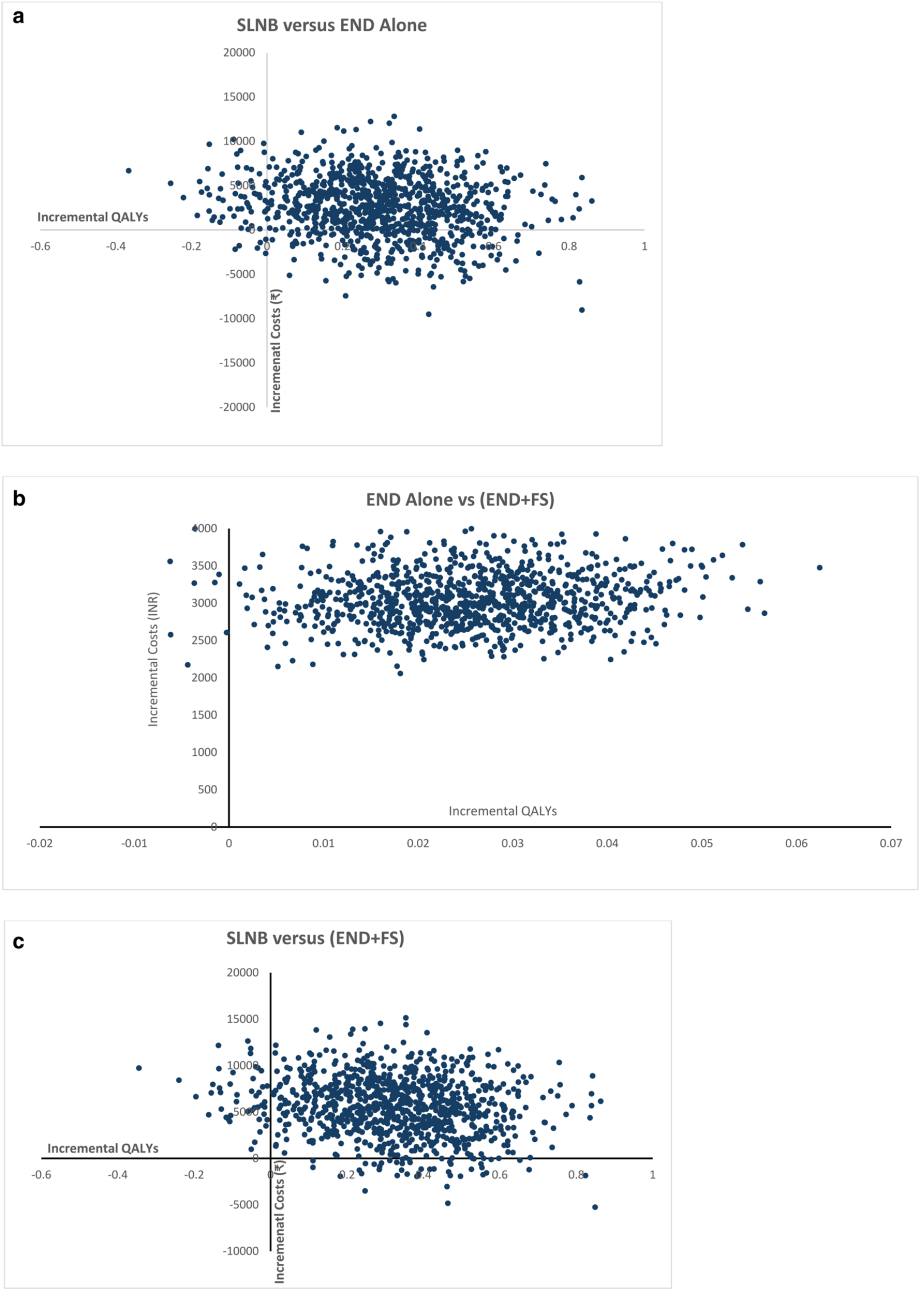
(a) Cost‐effectiveness plane showing the incremental costs and QALYs for SLNB guided neck dissection (Group I) vs. END Alone (Group II) over a lifetime horizon. (b) Cost‐effectiveness plane showing the incremental costs and QALYs for END Alone (Group II) vs. END with Frozen Section Analysis (Group III) over a lifetime horizon. (c) Cost‐effectiveness plane showing the incremental costs and QALYs for SLNB guided Neck Dissection (Group I) vs. END with Frozen Section Analysis (Group III) over a lifetime horizon.

**FIGURE 3:**
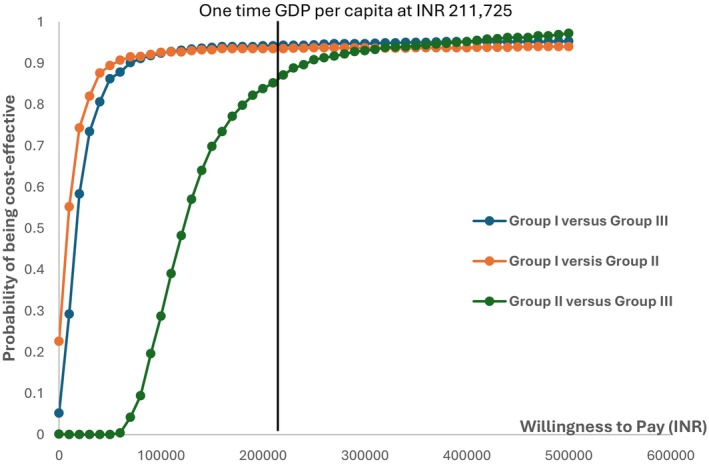
Cost‐effectiveness acceptability curves for comparison Group I vs. Group II, Group I vs. Group III and Group II vs. Group III. Group I: Sentinel Lymph Node Biopsy guided Neck Dissection; Group II: Elective neck Dissection Alone; Group III: Elective Neck Dissection with Frozen Section Analysis.

The cost‐effectiveness plane, displaying 1000 Monte Carlo simulations for incremental costs and QALYs gained, is shown in Figure 2a–c for Group I versus Group II, Group I versus Group III, and Group II versus Group III, respectively. Figure 3 presents the cost‐effectiveness acceptability curve, illustrating the probability of Group I versus Group II, Group I versus Group III, and Group II versus Group III being cost‐effective at 93.5%, 94.2%, and 85.2%, respectively, at the one‐time GDP per capita threshold.

## 4 Discussion

The present study demonstrates that SLNB guided neck dissection is a cost‐effective strategy as compared to END Alone/END with Frozen Section Analysis, in the management of patients with cT_1_T_2_ N_0_ oral cancer. In our model, the 5‐year OS among the patients undergoing END (with or without FS) was 78.98%, while that with the SLNB strategy was 79.32%. The DFS was noted to be the same in all strategies (71.82%). However, the QALYs of Group I were recorded to be 8.43, which was higher in comparison to 8.12 QALYs and 8.097 QALYs observed in Group II and Group III, respectively. Overall, in terms of survival, Group I was non‐inferior to Group II/III and was yet associated with lesser morbidity. These results are in concurrence with recently published meta‐analyses that reported comparable survival rates between the two strategies with better HRQoL, suggesting SLNB to be a superior alternative to the conventional END approach [7, 52, 53].

Our model presents SLNB to be a costlier strategy with an average cost of INR 181,520 (US$ 2169) per person in a lifetime. This is in contrast to the results of an observational study that reported the SLNB procedure to be 42% less expensive than END [54]. Another analytical modeling study that used data from the European Sentinel Node (SENT) trial reported that the cost of treatment with the SLNB pathway is 0.35–0.60 times lower than the cost associated with the END technique [55]. This variation in the costs estimated is because our model has considered the lifetime treatment costs including the cost of surgery and postoperative adjuvant treatment in contrast to the other studies that have included the surgical costs alone. Although SLNB adds on to the cost of treatment, it also yields higher QALYs than END, which is consistent with the other published studies [8, 9]. We understand that the cost‐effectiveness estimates may differ from one country/region to another due to various factors such as differences in the mortality rates, age structure, prevalence of the disease, the efficiency with which treatment for the particular disease in question is delivered, and importantly, the differences in the local costs of the services [56].

Considering our research findings, SLNB guided neck dissection is more expensive, but an effective strategy with an estimated 94% probability of being cost‐effective given the GDP per capita threshold. It is evident that performing the SLNB procedure demands substantial resources and precise execution. Among the prerequisites, having a fully equipped nuclear medicine department stands out as crucial, along with other essential resources necessary for the successful completion of the procedure. According to the most recent report by the Atomic Energy Regulatory Board, India has a total of 293 nuclear medicine departments, with 14% of them being government‐owned [57]. Additionally, it is noteworthy that the SLNB procedure is included in the *PM‐JAY* health benefit packages and is available in both private and public empanelled hospitals nationwide. Furthermore, the success of the SLNB procedure is greatly influenced by the proper training of medical personnel in the technique‐sensitive aspects of the procedure. Notably, the utilization report of oncology packages for *PM‐JAY* indicates that the top 10 facilities which contribute to 60% of the oncology claims are well‐equipped institutions with advanced facilities [42]. In light of the cost‐effectiveness of the SLNB procedure compared to the traditional neck dissection (i.e., END) and its feasibility in the Indian healthcare setting, we recommend considering SLNB guided neck dissection for early‐stage oral cancer patients.

It is important to note that our study is not specific to any sub‐site of oral cavity and this is a limitation of our model assumption. The site of oral cavity is a strong predictor of oral cancer prognosis, and the diagnostic accuracy of SLNB varies with the site, the sensitivity and NPV being more for buccal mucosa and less for floor of the mouth cancers. However, the estimate reported in the study by Toom et al. [22] is adjusted for this variation. Another limitation of our model is the assumption regarding similar transition probabilities of LRR in patients subjected to SND versus MND in the END with FS treatment arm. However, retrospective analysis of clinically node negative yet pathologically node positive OSCC patients reported no significant difference between the SND and MND groups on the disease‐specific survival rates [58]. Moreover, for patients undergoing adjuvant treatment following primary surgery, our model has not considered postoperative T stage, lympho‐vascular invasion, peri‐neural invasion, depth of invasion, grade of tumor, margin status, worst pattern of invasion as risk factors for disease progression. However, we did consider pathologically active lymph nodes as the prognostic risk factor which is established to be an independent predictor significantly associated with adverse outcomes of the disease [59, 60].

The strength of our model can be substantiated based on the assumptions made regarding several input parameters. Firstly, the health state utility values utilized in our calculations have been sourced from India's national database, CaDCQoL, which enhances the credibility and reliability of our effectiveness estimates [37]. Secondly, we have included the treatment cost data from the HBP 2022 estimates that are inclusive of the cost variations across the public‐private health care facilities across different cities in India. Moreover, considering the high survival rates among OSCC patients, we have computed our estimates for a lifetime horizon for a comprehensive assessment of both the costs and effects over the entire duration of a patient's life.

## 5 Conclusion

SLNB guided neck dissection helps avoid unnecessary overtreatment, leading to improved quality of life at reasonable costs and is a cost‐effective strategy as compared to END Alone/END with FS analysis. Moreover, there is potential for SLNB guided neck dissection to become a cost‐saving strategy by improving the diagnostic accuracy of the SLNB procedure.

## Author Contributions

**Shivakumar Thiagarajan:** conceptualization (equal), data curation (equal), visualization (equal), writing – review and editing (equal). **Shweta Sharda:** data curation (equal), formal analysis (equal), methodology (equal), validation (equal), writing – original draft (equal). **Yashika Chugh:** data curation (equal), formal analysis (equal), methodology (equal), validation (equal), writing – original draft (equal). **Nidhi Gupta:** methodology (equal), visualization (equal), writing – review and editing (equal). **C. S. Pramesh:** conceptualization (equal), methodology (supporting), writing – review and editing (equal). **Shankar Prinja:** conceptualization (equal), formal analysis (equal), methodology (equal), supervision (lead), writing – review and editing (equal).

## Funding

The authors have nothing to report.

## Conflicts of Interest

The authors declare no conflicts of interest.

## Supporting information


**Figure S1:** Tornado diagram of the univariate sensitivity analysis showing the impact of individual parameters on the incremental costs per QALY gained in SLNB guided neck dissection versus END with FS over a lifetime horizon.
**Table S1:** Health outcomes and costs incurred in the three treatment groups at a 5‐year time‐horizon.
**Figure S2:** Scatter plot showing the incremental costs and QALYs for SLNB guided neck dissection versus END+FS over a 5‐year time horizon.
**Table S2:** Health outcomes and costs incurred in the three treatment groups at a 10‐year time‐horizon.
**Figure S3:** Scatter plot showing the incremental costs and QALYs for SLNB guided neck dissection versus END+FS over a 10‐year time horizon.
**Figure S4:** Threshold analysis showing the breakpoint rate of occult metastasis till which SLNB guided neck dissection is cost‐effective at one‐time GDP per capita threshold for India.

## Data Availability

The data that support the findings of this study are available from the corresponding author upon reasonable request.
