# Supplementary material for: Sentinel Lymph‐Node Biopsy Guided Neck Dissection Versus Elective Neck Dissection in the Management of Early‐Stage Oral Cancer—A Cost‐Utility Analysis
Source: Cancer Med. 2026 Feb 5;15(2):e71571. doi: 10.1002/cam4.71571 (PMC12875840; doi:10.1002/cam4.71571)
Supplement: Supplementary file 1 — Figure S1: Tornado diagram of the univariate sensitivity analysis showing the impact of individual parameters on the incremental costs per QALY gained in SLNB guided neck dissection versus END with FS over a lifetime horizon. Table S1: Health outcomes and costs incurred in the three treatment groups at a 5‐year time‐horizon. Figure S2: Scatter plot showing the incremental costs and QALYs for SLNB guided neck dissection versus END+FS over a 5‐year time horizon. Table S2: Health outcomes and costs incurred in the three treatment groups at a 10‐year time‐horizon. Figure S3: Scatter plot showing the incremental costs and QALYs for SLNB guided neck dissection versus END+FS over a 10‐year time horizon. Figure S4: Threshold analysis showing the breakpoint rate of occult metastasis till which SLNB guided neck dissection is cost‐effective at one‐time GDP per capita threshold for India. [file CAM4-15-e71571-s001.docx]

**ICUR at base value: INR 16,709**

*Note: The parameters are arranged in the decreasing order of their impact on the ICER values. The parameters not reported on the tornado diagram had no significant effect on the ICUR values.*

*SLNB: Sentinel Lymph Node Biopsy; LRR: Loco-regional recurrence; DFS: Disease-free-survival.*

**Figure S1: Tornado diagram of the univariate sensitivity analysis showing the impact of individual parameters on the incremental costs per QALY gained in SLNB guided neck dissection versus END with FS over a lifetime horizon.**

**Table S1: Health outcomes and costs incurred in the three treatment groups at a 5-years time-horizon.**

| **Treatment Groups** | **QALYs per person** | **Cost per person**  **INR (US $)** | **Incremental QALYs** | **Incremental costs** | **Status** |
| --- | --- | --- | --- | --- | --- |
| Elective Neck Dissection with frozen section analysis  **(Group III)** | 3.49 | INR 80,389  (US $ 961) | - | - | - |
| Elective Neck Dissection alone  **(Group II)** | 3.52 | INR 84,775  (US $ 1,013) | 0.03 | INR 4,386  (US $ 52) | - |
| Sentinel Lymph Node Biopsy guided neck dissection  **(Group I)** | 3.66 | INR 72,599  (US $ 868) | 0.14 | -INR 12,176  (-US $ 146) | **Dominant strategy** |

*QALYs: Quality-adjusted-life-years; NMB: Net Monetary Benefit*

*Note: This table represents the results of dominance analysis which is based on the deterministic results. The treatment groups are ordered in terms of increasing effects (QALYs). The negative sign denotes cost savings.*

**Figure S2: Scatter plot showing the incremental costs and QALYs for SLNB guided neck dissection versus END+FS over a 5-year time horizon.**

**Table S2: Health outcomes and costs incurred in the three treatment groups at a 10-years time-horizon.**

| **Treatment Groups** | **QALYs per person** | **Cost per person**  **INR (US $)** | **Incremental QALYs** | **Incremental costs** | **Status** |
| --- | --- | --- | --- | --- | --- |
| Elective Neck Dissection with frozen section analysis  **(Group III)** | 5.67 | INR 121,603  (US $ 1,453) | - | - | - |
| Elective Neck Dissection alone  **(Group II)** | 5.70 | INR 124,725  (US $ 1,491) | 0.03 | ₹3,132  (US $ 37) | **-** |
| Sentinel Lymph Node Biopsy guided neck dissection  **(Group I)** | 5.86 | INR 117,126  (US $ 1,340) | 0.16 | -₹7,599  (-US $ 91) | **Dominant strategy** |

*QALYs: Quality-adjusted-life-years; NMB: Net Monetary Benefit*

*Note: This table represents the results of dominance analysis which is based on the deterministic results. The treatment groups are ordered in terms of increasing effects (QALYs).*

**Figure S3: Scatter plot showing the incremental costs and QALYs for SLNB guided neck dissection versus END+FS over a 10-year time horizon.**

**Cost-Effective**

**Cost-effectiveness Threshold for India = one-time GDP per capita**

**Not Cost-Effective**

**Figure S4: Threshold analysis showing the breakpoint rate of occult meta-stasis till which SLNB guided neck dissection is cost-effective at one-time GDP per capita threshold for India.**
